# Human Papillomavirus (HPV) virion induced cancer and subfertility, two sides of the same coin

**Published:** 2016-12

**Authors:** CE Depuydt, J Beert, E Bosmans, G Salembier

**Affiliations:** Department of Clinical and Molecular Pathology, AML, Sonic Healthcare, Antwerp, Belgium; Intermediate structure human body material, AML, Sonic Healthcare, Antwerp, Belgium

**Keywords:** infertility, clonal, transient virion producing, spermatozoa, semen, donor sperm

## Abstract

In the natural history of HPV infections, the HPV virions can induce two different pathways, namely the infec- tious virion producing pathway and the clonal transforming pathway. An overview is given of the burden that is associated with HPV infections that can both lead to cervical cancer and/or temporal subfertility. That HPV infections cause serious global health burden due to HPV-associated cancers is common knowledge, but that it is also responsible for a substantial part of idiopathic subfertility is greatly underestimated. The bulk of the detected HPV DNA whether in men or women is however infectious from origin. Because the dissociation between HPV viruses and HPV virions or infection and disease remains difficult for clinicians as well as for HPV detection, we propose a review of the different effects caused by the two different HPV virion induced pathways, and highlight the mechanisms that are responsible for causing transient subfertility and cancer.

## Introduction

**Fig. 1:**
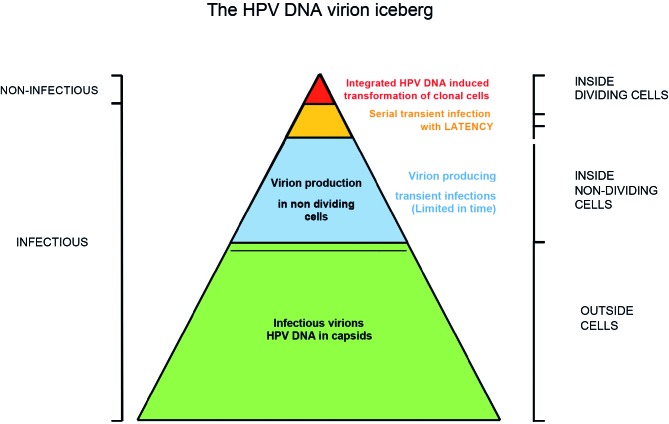
— The HPV DNA virion iceberg. Green: Largest HPV DNA fraction inside L1-L2 protein capsid, free outside cells and infectious; Blue: HPV DNA present in transient virion producing infections inside non-dividing desquamating cells, limited in time, infectious; Red: Smallest HPV DNA fraction comprising of integrated HPV DNA inside dividing cells, HPV transforming pathway can lead to cancer but is non-infectious; Orange: HPV DNA present in both non-dividing virion producing cells and in transformed dividing cells, both the virion producing and clonal HPV transforming pathways occur simultaneously, can lead to cancer and is infectious.

For almost two decades it has been recognized that infection with human Papillomavirus (HPV) is one of the major causes of infection-related cancer worldwide (Bosch and Munoz, 2002; Walboomers et al., 1999), as well as the causal factor in other diseases (Bosch et al., 2013). For both men and women there is strong evidence for a causal aetiology with HPV for cancers of the cervix uteri, penis, vulva, vagina, anus and head and neck cancers (Bosch et al., 2013; Tomaic, 2016; Walline et al., 2013). Of the more than 10 million new cancers occurring each year worldwide, it is estimated that 4.8% were attributable to HPV infection. The limited amount of viral HPV DNA inside cancer cells however only represent a minute fraction of the total HPV DNA present in men and women. The bulk of HPV DNA is present in the virions (Fig. 1), the infectious messenger that probably almost every human being on this planet had the privilege of harbouring at one moment in his life. Like an iceberg the viral DNA in the infectious lifecycle part of the virus remains unnoticed and its global burden of HPV and related diseases remain largely underestimated.

Still today there is debate whether a virus is alive or not and this is largely due to semantic differences in the definition of what a virus is or which characteristics are needed to describe its biological entity. It was only at the end of last century that Bandea proposed the idea that virus and virion are distinct entities (Bandea, 1983). According to Bandea’s hypothesis, the “HPV infected cell” is the virus, while the virus particles or virions are “spores” or reproductive forms. In analogy with Janus a god of beginnings and endings, a virus is an organism with two phases, the virion and the infected cell. Although it is easy to understand that viruses are organisms that pass in their ontogenetic cycle through two distinct phenotypic phases, the dissociation between viruses and virions or infection and disease remains difficult for clinicians and HPV detection.

In the vegetative state or mature phase of the viruses, their component molecules are dispersed within the host cell. There is less debate for the infectious particles or virions not being viruses. The infectious particle is designed for the transmission of nucleic acid genome among host or host cells and for HPV the problem lies in the fact that DNA based tests detect both virus and virions since they contain the same viral DNA. Because viral HPV DNA can be present in both virions and virus and in both non- dividing virion producing cells as well as in HPV viral transformed dividing cells. The distinction on a molecular base can be hard to make, the effects however are quite different. If we accept that the virus is the infected cell, then it becomes obviously clear that pathologists and clinicians have confused the virion and the virus.

**Fig. 2:**
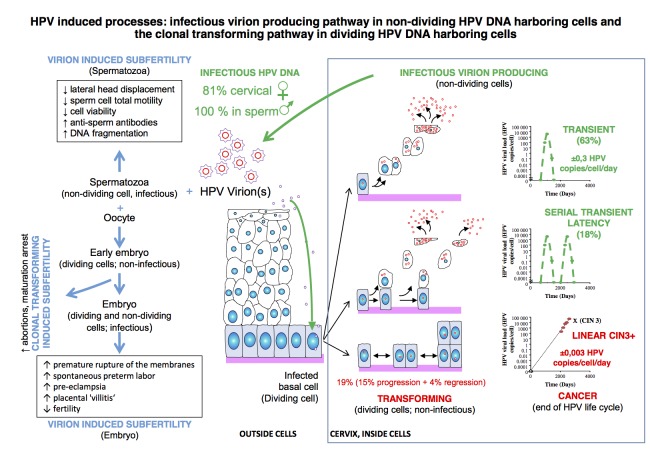
— Overview of HPV induced processes defined on the basis of dividing and non-dividing HPV DNA harbouring cells.

By dividing the ontogenetic HPV cycle into its different phenotypic phases we demonstrate that the HPV virions lie at the bases of both temporal subfertility and in some rare cases of cancer (Fig. 2).

## HPV DNA tests detect both virus and virion

A good example of confusing virus and virion is HPV DNA measurement. HPV is a circular double stranded DNA virus comprising 6 early (E1, E2, E4, E5, E6 and E7) and 2 late genes (L1 and L2) and when only a part of the viral genome is measured it becomes even more difficult to separate virions from viruses. The expression of E6 and E7 oncogenes is required for maintaining the malignant growth of cervical cancer cells, specifically by inhibiting the tumour suppressors p53 en RB (Hausen, 2002). They are independently able to immortalize various human cell types in culture, but efficiency is increased when E6 and E7 are expressed together (Munger et al., 1989). Therefore HPV tests that measure in E6 or E7 are well suitable for detection of HPV in clonal transformed cells that can lead to cancer but will also detect the E6/E7 DNA part present inside virions. HPV tests that measure in L1 on the other hand are less suitable to detect viral DNA in clonally transformed cells (Tjalma and Depuydt, 2013) because upon entry of the initial virion in the first infected cell, the circular viral HPV DNA has to open and linearize to integrate into the host DNA (Pett and Coleman, 2007) to be able to transform the host cell and to start viral replication in the nucleus. Upon linearization and integration, the L1 gene can be disrupted and is at that moment not detectable with a HPV L1 test. Cells with the ability to divide that contain viral HPV DNA are unable to build and assemble new virions and this independently of possible integration via L1/L2. HPV tests detecting L1 are however very well suited to detect virions because the HPV L1 gene is always present. Since L1 and L2 proteins are needed to build the viral capsid of the virions.

Detection of HPV RNA does not allow to distinguish between virion and virus either because part of the double stranded HPV DNA can denature to single strand DNA and be picked up by RNA tests since the HPV RNA and DNA sequence are identical (Boulet et al., 2010).

Because none of the current HPV tests take into consideration the origin of the detected HPV DNA, the specificity of HPV based tests to detect disease is low, and triage of HPV positive women remains a problematic issue (Rebolj et al., 2014).

We therefore proposed to use serial type specific viral load measurements (≥ 3) to differentiate between transient virion producing HPV infections and HPV transformed clonal cell populations (Depuydt et al., 2012; Depuydt et al., 2015, 2016a; Verhelst et al., 2016).

## Transient HPV virion producing non-dividing cells and clonally HPV transformed cell populations

That the HPV infectious life cycle is closely linked to the differentiation state of the stratified epithelium it infected, has been known for some time (Stubenrauch and Laimins, 1999). The HPV life cycle differs from other virus families, i.e. infection requires the availability of epidermal or mucosal cells that are still able to divide, namely the basal layer cell (Hausen, 1996). Another crucial step is that HPV must first establish its infection within these dividing cells (Pyeon et al., 2009). In normal stratified epithelia, the only actively dividing cells are located in the basal and parabasal layers adjacent to the basement membrane and consist of stem cells as well as cells of limited lifespan called transit amplifying (TA) cells (Klumpp et al., 1997). As normal basal cells divide, one daughter cell becomes a new basal cell, while the other migrates away from the basal layer and begins to differentiate. Differentiating cells leave the proliferative cell cycle and undergo a complex series of changes in gene expression, eventually resulting in cell death and desquamation.

First the HPV virion infects cells of the poorly differentiated, proliferative, basal compartment of stratified epithelia. In these cells with dividing capacity, the viral genome sets up residence as a low-copy nuclear plasmid, with only a subset of viral genes (early genes) expressed at low levels, and no virions are made. HPV virions are very infectious, with a very low multiplicity of infection, 1-10 viral genome equivalents per cell is sufficient to establish infection (Patterson et al., 2005). The recipient cell must enter M phase (mitose) for HPV infection to take place (Pyeon et al., 2009). However when infected basal cells divide and non-dividing daughter cells migrate into the suprabasal compartment for terminal differentiation, the productive phase of the viral life cycle is initiated (Hausen, 2002). We previously showed 3 possibilities for HPV infected basal cell to divide, with a) division of the infected cell into two non-dividing differentiated cells and ultimately leading to new virion production and release in these non-dividing cells, b) asymmetric replacement with division leading to one parabasal and one basal cell and c) with division leading to two basal cells which both retain the possibility to divide (clonal HPV transformed pathway) (Depuydt et al., 2015). We showed that these 3 different ways of cell division can lead to only five different viral load courses, representing the two distinct HPV induced pathways, when type-specific viral load is measured (Depuydt et al 2012, 2015, 2016a) (Fig. 2).

**Fig. 3:**
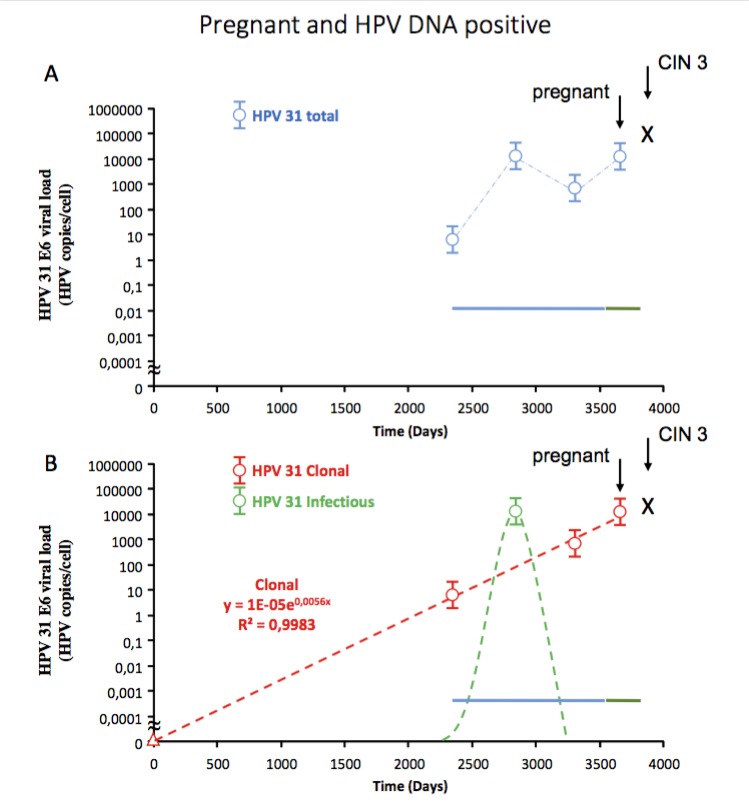
— Serial (linear time; x-axis) type specific HPV 31 E6 viral load measurements (logarithmic; y-axis) on liquid based cytology leftover in a 29 year old pregnant woman with CIN3. The solid blue line represents the period when patient tried to conceive, and the solid green line represents the duration of her pregnancy, x = detection of CIN3. A. Blue circles represent total measured HPV 31 viral load while conceiving, during pregnancy and prior to detection of clonal CIN3. B. Separation of the total measured HPV 31 load into clonal (red circles) and virion producing (green circle; measurements during transient phase). Red circles = measurement in the linear clonal transforming phase with R2 > 0.9. Red triangle represents the calculated starting point of the linear increase leading to CIN3 with viral load of 0.00001 HPV copies/cell. Dashed lines represents the transient course (green) and the linear course (red) of the infection between the viral load measurements. The woman only became pregnant after termination of the infectious virion producing phase (green) and when HPV 31 DNA was detectable in clonal transformed cells (non-infectious). Persistence of HPV 31 prompted treatment after pregnancy.

Although the infectious virion producing and clonal cell-transforming pathway can occur simultaneously at a single location (e.g. cervix) in patients (Depuydt et al., 2012, 2015, 2016a; Verhelst et al., 2016), it is in separate cells at different physical locations within the epidermis or mucosa. This vital aspect is often overlooked and contributes largely to the inability to discern between the two pathways and the ramifications that follow. A good example is that women presenting with low- grade intraepithelial lesions (L-SIL) on cervical cytology are referred to colposcopy because a small percentage has underlying cervical cancer. L-SIL cells detected in cervical cytology are the hallmark of HPV virion production and the halo that is visible microscopically represents the accumulation of newly formed virions within the differentiated non- dividing cell. A terminally differentiated dying cell that will desquamate can impossibly be a cancer cell. The clonal dividing cancer cell population if present is then located in the vicinity. Both the virion producing and clonally transformed cells can harbour the same HPV virus type and be measured using type specific quantitative qPCR (Depuydt et al., 2012, 2015, 2016a; Verhelst et al., 2016) (Fig. 3). An additional problem lies in the fact that cytological abnormalities caused by virion production are quickly visible after 3 to 4 weeks (Burd, 2016), whereas the clonal transformation by oncogenic HPV is very slow and the cytological abnormality that triggers biopsy taking or conisation is performed on average only after 484 weeks (Depuydt et al., 2012, 2016a). Needless to say that detection of viral HPV DNA can be confusing at best and triage of women with L-SIL can be problematic.

To add to the confusion, both the HPV virion producing and HPV transforming pathways can occur simultaneously, and be caused by the same HPV type. Because virtually none of the previous studies make a distinction from where the detected viral HPV DNA originates from, this creates a bias and leads to detection of viral HPV DNA in both normal and diseased conditions (Fig. 3).

For more than a quarter of a century it has been known that in benign and low-grade cervical intraepithelial (CIN) lesions, HPV DNA is present in episomal form that can be assembled into virions, whereas in the majority of the cervical tumours it is integrated into the host cell genome (Fig. 2) (Cullen et al., 1991).

## The HPV virion producing pathway in non- dividing desquamating cells can cause temporal subfertility

The sole purpose of virion production is to secure the survival of the HPV organism. By producing massive amounts of new virions in non-dividing cells, HPV ensures that it can infect new target cells.

Ironically in the case of temporal subfertility, early reports start with the detection of HPV in amniotic fluids from pregnant woman with cervical lesions (Armbruster-Moraes et al., 1994). It is in the beginning of the 90's that the first studies appear describing cervical HPV infection as a possible cause of early abortion (Manavi et al., 1991,1992). Material from spontaneous abortions was also found to be HPV infected at a significantly higher frequency than material from elective abortions (60% vs 20%, respectively) (Hermonat et al., 1997), and the main target of HPV induced abortion seemed to be the trophoblast (Hermonat et al., 1998).

Although a number of studies followed showing that an infection with HPV during pregnancy is associated with the risk of spontaneous abortion (Hellberg and Nilsson, 2007), premature rupture of the membranes (Cho et al., 2013), spontaneous preterm delivery (Gomez et al., 2008; Zuo et al., 2011), pre-eclampsia (McDonnold et al., 2014), and placental ‘villitis’ (Slatter et al., 2015; Zuo et al., 2011), HPV testing is not performed in women seeking to conceive. Others studies have detected HPV DNA with widely ranging percentages from 6% to 65% and with controversial results. There have been a number of studies that suggested that HPV infection may be found commonly in placental material and might be linked with spontaneous abortions (Armbruster-Moraes et al., 1994; Favre et al., 1998; Hermonat et al., 1997,1998; Malhomme et al., 1997; Manavi et al., 1991; Pao et al., 1995; Rabreau and Saurel, 1997; Sikstrom et al., 1995). However in a cross-sectional study performed in Poland on placentas from women with spontaneous abortions and from control women after term delivery no association between HPV 16/18 and miscarriages was observed (Skoczynski et al., 2011). Unfortunately nothing is known about other HPV types in this study, and HPV 16/18 could have caused spontaneous abortions before the 6th week of pregnancy due to their more oncogenic potential compared to other high-risk HPV types. That HPV 16/18 induces early stage-specific maturation arrest in infected mouse embryos has previously been shown (Henneberg et al., 2006). A recent review article, with pooled data demonstrated an association between spontaneous abortion, spontaneous preterm delivery and the presence of HPV in both the cervix and the placenta (Ambuhl et al., 2016).

Also here it is clear that both HPV virion and HPV virus have been measured simultaneously and has resulted in apparently contradictory results confusing the role of HPV in abortion. For some papers/authors the study on HPV detection on pregnant women (Eppel et al., 2000) argues against involvement of HPV in abortion, but none of the pregnant women had miscarriages. However a retrospective study showed that HPV positive women undergoing intra uterine insemination (IUI) were six fold less likely to become pregnant compared to HPV negative women (1.87% vs 11.36%) (Depuydt et al., 2016b). Also for in vitro fertilization a significantly decreased pregnancy rate in women with HPV infection was reported compared to those who were HPV negative (23% vs 57%) (Spandorfer et al., 2006). One of the common misconceptions when detecting HPV DNA in women is that it does not preclude from achieving a pregnancy. It only diminishes the chances with increasing amount of free virions being able to bind or to enter the oocyte. For pregnant women when detecting oncogenic HPV DNA one should make sure that the viral DNA does not originate from a clonal HPV transformed cell population that could develop into cancer (Fig. 3). Women with HPV induced cervical cancer without simultaneously occurring HPV virion-producing infection would not be at increased risk for miscarriages or have reduced chances of achieving a pregnancy because no HPV virions are present, and the detected HPV DNA is confined to the nucleus of the dividing HPV transformed cells. As shown by a recent study on a large group of 2462 women that were followed for up to 10 years, none of the 122 women with a history of pregnancy during follow-up had L-SIL and no association between HPV and pregnancy was found (Trottier et al., 2015). A single case of high grade SIL occurred post-partum but the authors failed to see the connection between the two HPV induced pathways.

In studies were the localization of the HPV DNA was also performed, it is clear that trophoblast are involved (Hermonat et al., 1998). Trophoblasts are differentiated non-dividing cells that allow virion production and assembly. Trophoblasts are the cell type that maintains placental contact with maternal tissue and through which nutrient exchange occurs.

Ironically it is these kind of cells that were used to describe the HPV 16 and HPV 31 life cycle (You et al., 2003, 2008) and eventually led to the creation of virus like particles (VLP) used in current vaccines that protect against cervical, vaginal, vulvar, and anal cancers and genital warts caused by different types of HPV.

## HPV sperm infections

Recently a growing number of evidence linked to sperm concurred to identify the impairment of human reproduction as a consequence of HPV infection (Foresta et al., 2015b; Souho et al., 2015).

According to literature there is a huge difference of the most important clinical consequence of high-risk HPV infection in women versus men. In women it remains one of the leading causes of cancer-related mortality in young and older women worldwide. Furthermore it was recently estimated that among the 12.7 million new cancers occurring in women in 2008 worldwide, 700 000 cases occurred at an HPV- associated cancer site, and 610 000 of these were attributable to HPV (Bosch et al., 2013). This is in contrast to the rare HPV-related cancers at penile, anal, and oropharynx sites in male estimated to occur in only 1-6/100 000 in the general population (Giuliano et al., 2015).

Because of absence of dividing cells in sperm, the viral HPV DNA that is detected mainly originates from virions or from terminally differentiated (non- transformed) cells that are producing virions, for women however a substantial amount of HPV DNA detected at the cervix has a clonal origin (Fig. 2) and leads to cancer. Also HPV sero-prevalence is significantly higher among women compared to men, likely explaining the differences in age-specific HPV prevalence and incidence patterns observed by gender. Correspondingly, among heterosexual partners, HPV transmission appears higher from women to men (Giuliano et al., 2015). Another difference is the median duration of HPV infection, in men it was only 6.9 months (Kreimer et al., 2013) and 9.8 months in women (Rositch et al., 2013), although for women no distinction is made if the detected HPV infection was virion producing or clonal.

A recent meta-analysis and systematic review of HPV prevalence in semen identified 27 studies reporting HPV DNA prevalence in 4029 semen samples varying from 0 to 100% (Laprise et al., 2014). For the seven studies focusing on infertile populations the HPV prevalence was 16% (95% confidence interval (CI): 10-23%). Although oncogenic HPV DNA was measured no reference is made on how many penis cancers were detected. This might again suggest that virion and virus has been confused and that the detected HPV DNA mainly originates from virions of the infectious cycle. Comparing fertile with infertile patients, a higher HPV prevalence has been shown in semen of infertile patients in several studies. Interestingly, in fertile control subjects a lower HPV prevalence was observed in semen (Foresta et al., 2010b; Olatunbosun et al., 2001; Yang et al., 2013). When only transient virion producing HPV infections are considered, although in some men HPV sperm infections can be long lasting (24 months), the mean clearance is estimated to be six times shorter than in women (5.9 months vs 28.0 months) (Depuydt et al., 2012; Lenzi et al., 2013). Some authors reported that HPV infections are frequently associated with anti-sperm antibodies that may further reduce male fertility (Garolla et al., 2013). Over the years several studies tried to wash off HPV DNA from sperm without success (Brossfield et al., 1999; Chan et al., 1994; Olatunbosun et al., 2001), suggesting either a very strong binding of the virion to a receptor on the sperm cell surface or an entrance of HPV into the sperm cell. The presence of HPV virions that can bind two distinct sites along the equator of the spermatozoa’s heads (Foresta et al., 2010a; Garolla et al., 2013; Kaspersen et al., 2011; Perez-Andino et al., 2009) may not only have a detrimental effect on sperm parameters (Didelot-Rousseau et al., 2007; Foresta et al., 2010a; Garolla et al., 2012; Kaspersen et al., 2011; Lai et al., 1997; Rintala et al., 2004; Yang et al., 2013) but also on gamete interaction, since in vitro experiments have demonstrated that spermatozoa can not only transfer HPV virions (Foresta et al., 2011b) to the oocyte, but the transferred HPV virions also induce stage-specific maturation arrest in infected embryos (Henneberg et al., 2006). Because the HPV virions can physically bind the head of spermatozoa both the amount of virions present and sperm concentration will probably influence the chance to fertilize an oocyte with a virion negative spermatozoa. Even when HPV virions are detected in sperm pregnancies can still be achieved, albeit less frequent and with a greater risk for miscarriages (Ambuhl et al., 2016).

HPV infection can exerts its detrimental effects in different ways through its virions. The free virions can bind the spermatozoa’s head and directly reduce sperm motility, and while some find DNA strand breakages in spermatozoa exposed to HPV E6/E7 fragments (Connelly et al., 2001) others do not find HPV to induce sperm DNA damage (Garolla et al., 2012; Kaspersen et al., 2013). As some HPV types are more oncogenic than others, an HPV type specific effect on spermatozoa cannot be ruled out. When virions attached to spermatozoa encounter anti HPV IgG at the cervix in partners with immunity this could lead to agglutination and immobilization of spermatozoa preventing fertilization.

Since 1994 a growing number of studies demonstrated a possible correlation between HPV sperm infection and cases of idiopathic asthenozoospermia and unexplained infertility (Brossfield et al., 1999; Chan et al., 1994; Connelly et al., 2001; Foresta et al., 2010a, 2010b, 2011a, 2011b; Garolla et al., 2012, 2013; Gizzo et al., 2014; Lai et al., 1997; Lee et al., 2002; Rintala et al., 2004; Schillaci et al., 2013).

## HPV virion point of entry?

Almost 20 years before HPV virions were shown to attach to two distinct sites at the spermatozoon’s head (Foresta et al., 2010a, 2011b; Kaspersen et al., 2011), radiolabeling methods showed that absorbed exogenous DNA fragments localize to the postacrosomal and equatorial regions of the sperm head (Francolini et al., 1993), while the remaining excess DNA fragments are localized externally at the membrane surface, making it possible to transfect the plasma membrane of embryonic cells at the time of contact (Chan et al 1995a, 1995b). That spermatozoa have the ability to transfer HPV virion(s) into the oocyte has been known for a decade (Henneberg et al., 2006), the effect of introducing an infectious particle into a dividing embryo has been observed for more than two decades (Manavi et al., 1991). In the beginning the HPV virion will set up residence as a low-copy nuclear plasmid, a subset of viral genes (early genes) are expressed at low levels, and no progeny virus (virion) is made. Probably depending on the HPV type (Chan et al., 1995b) and its oncogenic potential (high risk, intermediate risk) the clonal expansion can be abruptly stopped or lasts just a little bit longer to allow the embryogenesis to start differentiation. In the beginning there are only dividing cells and these cells do not allow virion production. When however the clonal expansion of HPV harbouring cells stop and these cells begin to differentiate, the viral DNA will be able to make L1 and L2 proteins and produce new virions. Unluckily for the embryo, it needs differentiated cells to permit implantation into the endometrium but these differentiated syncytiotrophoblast now allow the productive stage of the viral life cycle to be initiated and start making massive amounts of new virions. As shown by a previous study at the end of last century, trophoblasts are the preferential target for HPV infection in spontaneously aborted products of conception (Hermonat et al., 1998). Accumulation of HPV virions in the trophoblasts that are responsible for maintaining placental contact with maternal tissue and through which nutrient exchange occurs, prompted the hypothesis that HPV-infected trophoblasts may have altered characteristics which may lead to a compromised gestation (Hermonat et al., 1998; You et al., 2002).

A number of HPV types are found in trophoblasts of spontaneous abortions and replicate in these cells in culture (You et al., 2008). Infection with HPV 16 and HPV 16 oncogene expression may not only lead to outright trophoblast death, but to defective endometrial cell recognition or even malignant phenotype (Liu et al., 2005; You et al., 2002). Beside HPV 16, multiple other HPV types have been shown to replicate in trophoblasts (You et al., 2003, 2008).

The HPV virion or HPV DNA can enter the oocyte directly by hitchhiking with the first spermatozoa that enters the oocyte, but it has also been shown that life spermatozoa that absorbed HPV 16 and HPV 18 E6/E7 DNA can transfer this viral DNA into both the inner cell mass as well as to the trophoblast cells of mouse blastocyst (Cabrera et al., 1997; Chan et al., 1995b). It is thus possible that even after fertilization of the oocyte HPV DNA can still enter at the later blastocyst stage thru the remaining HPV virion carrying spermatozoa.

The chorionic villi emerge from the chorion, invade the endometrium, and allow transfer of nutrients from maternal blood to foetal blood. The chorion consists of two layers: an outer formed by the trophoblast, and an inner formed by the somatic mesoderm; the amnion is in contact with the latter. The trophoblast is made up of an internal layer of cubical or prismatic cells, the cytotrophoblast or layer of Langhans, and an external layer of richly nucleated protoplasm devoid of cell boundaries, the syncytiotrophoblast. A recent clinicopathological study of HPV infection of the human placenta and pregnancy complications showed that HPV was found in the decidua of 75% of placenta (253/339) and was associated with histological acute chorioamniotis (Slatter et al., 2015). The study also showed that in 14% (35/253) of the HPV positive cases, HPV L1 immunoreactivity also occurred in the villous trophoblast where it was associated with a lymphohistiocytic villitis (HPV-LHV), and was exclusively of high-risk HPV type. HPV-LHV was also significantly associated with foetal growth restriction, preterm delivery, and pre-eclampsia.

It is clear that in cervical HPV infection during pregnancy both the virion producing and clonal transforming pathways are involved. Whereas premature rupture of the membranes (Cho, G. et al., 2013), spontaneous preterm labour (Gomez et al., 2008; Zuo et al., 2011), pre-eclampsia (McDonnold et al., 2014), and placental ‘villitis’ not otherwise specified (Slatter et al., 2015) is a burden due to the virion producing pathway. The higher incidence of spontaneous abortions (Hellberg and Nilsson, 2007; Hermonat et al., 1997; Malhomme et al., 1997), can probably be ascribed to the division stop induced by the presence of HPV oncogenes of the transforming pathway.

## HPV vaccination a possible therapy to prevent and shorten the HPV infectious cycle?

A recent study showed that prophylactic HPV vaccination in men improves the clearance of HPV semen infection (Foresta et al., 2015a).

That the transformation of a clonal cell population by the E6/E7 HPV oncogenes can be detrimental for the patient is evident. But also for the HPV virus it means the end of its life cycle since dividing cells do not allow to produce infectious progeny. That the small fraction of HPV DNA present in cervical and other HPV induced cancers are self-limiting and do not allow infectious spreading of HPV viruses did not stop the HPV viruses from being intimate with humans for the last 500 000 years. This half million year old relationship argues against resolving HPV infections as a result of a cell-mediated immune response because the interaction between the virus and the host's immune system strongly influences viral evolution (Holmes, 2008).

Papillomaviruses (PV) are well adapted to their host, and can in most instances complete their life- cycle and be maintained in the population without causing any apparent disease (Ekstrom et al., 2011; Forslund, 2007). Such characteristics suggest that the PV-host interactions are very old, and that over time, this has led to a balance between viral replication and immune tolerance (Woolhouse and Gaunt, 2007). The evolutionary origins of PVs can be traced back to approximately 350 million years ago, at the origin of the amniotes themselves (Garcia-Vallve et al., 2005; Gottschling et al., 2007; Shah et al., 2010).

Looking at the overview of HPV induced processes, defined on the basis of dividing and non-dividing HPV DNA harbouring cells, a strong immune response would be surprising considering the scare contact of virions with the host immune system. Unlike other viruses, HPV does not infect or replicate in antigen-presenting cells of the epithelium nor induce cell lysis, so there is no chance for antigen-presenting cells to present antigens derived from the virion to the immune system. In real life this results in seroconversion of only 50% of infections present in the patients, the production of antibodies usually occurs only months after the initial infection (Tindle, 2002), and some women with type-specific persistent HPV infection even fail to seroconvert (Buchinsky et al., 2006; Carter et al., 2000). The injection of HPV virus like particles directly into the body when vaccinating probably explains the efficacy of HPV prophylactic vaccination with Gardasil in men to improve the clearance of HPV semen infection (Foresta et al., 2015a). Also early data from Australia are already showing high efficacy in prevention of genital warts in immunized cohorts of females and significant but lower efficacy in unimmunized males from the same population, a significant example of the impact of herd immunity (Bosch et al., 2013). By targeting specific HPV types such as HPV 6, 11, 16 and 18 by vaccination the prevalence of these HPV types is dropping quickly in vaccinated populations (Depuydt et al., 2013) and could be extinct quickly after the introduction of the vaccines less than 10 years ago. In Belgium vaccination significantly reduced the prevalence of HPV 11 by 48,9% and HPV 16 by 18,6% in the period from 2006 till 2012 (Depuydt et al., 2013).

## Treatment of HPV and effect on pregnancy

A recent systematic review and meta-analysis on fertility and early pregnancy outcomes after treatment for CIN showed a higher overall pregnancy rate for treated women than for untreated women (four studies; 43% v 38%, pooled relative risk 1.29,95% confidence interval 1.02 to 1.64;P<0.0001) (Kyrgiou et al., 2014). Suggesting that the removal of the HPV virion production site at the cervix improves the pregnancy rate.

## HPV testing of donor sperm

Considering the incomplete data of the HPV effect on fertility and conflicting results from studies with control groups showing an effect of HPV on sperm parameters and studies without control groups unable to show an HPV effect on measured sperm parameters (Golob et al., 2014; Luttmer et al., 2016; Rintala et al., 2004; Schillaci et al., 2013), it is not surprising that not everybody is convinced that standardized HPV testing of semen should be performed in the diagnostic work-up of subfertile couples. Hopefully new studies with control groups assessing the effect of the measured HPV on fertility outcome can in a not too distant future show a deleterious effect on fertility. However what all studies agree upon is that oncogenic HPV DNA can be measured and detected in semen, that a detrimental effect of seminal HPV on early embryo development and clinical reproductive outcomes cannot be excluded and that screening of donor semen for HPV should be considered to prevent iatrogenic cervical HPV infections in recipient women. Notwithstanding the fact that the multiplicity of infection for HPV virions is very low and minute amount of virions are sufficient to infect target cells and interact with spermatozoa and embryos, it is the exception and the termination of HPV life cycle in clonal populations that drive to perform HPV testing of donor sperm. Only a handful of studies published HPV prevalence in sperm from donors (Kaspersen et al., 2011; D’Hauwers et al., 2012) and even less recommend that sperm banks and donors should be tested for HPV (Basky, 2000; Foresta et al., 2011a). Although the sperm donors are labelled healthy, a percentage is positive for oncogenic HPV types and no correlation was made on how often or how frequently these donors were successful to achieve pregnancies compared to the healthy HPV negative sperm donors (Kaspersen et al., 2013).

From our own data from a prospective study on HPV typing of 142 semen samples from 62 sperm donors, on the residual semen material of the semen sample used on the day of insemination, only 3 from the 62 donors were positive for oncogenic HPV (4.84%). None of the 7 inseminations from these HPV positive donors resulted in a pregnancy in any of the 5 women who underwent an intra uterine insemination on the day of ovulation. In the remaining 135 inseminations, 20 pregnancies were achieved. In the group of IUI with husband/partner semen 13.85% (59/426 IUI cycles) were performed with high-risk HPV positive semen. Based on this observation it could be highly beneficial for assisted reproductive technology methods if one could establish a link between the presence of a HPV infection and a significant decrease in the success rate of IUI. According to the results report generated from European registers by ESHRE for the year 2012 (Calhaz-Jorge et al., 2016) a total of 175.028 IUI with husband/partner semen and 43.497 IUI with donor semen have been performed. Based on our observed frequencies, this would lead to 175.028 x 0.138 HPV infected semen samples in partner IUI and 43.497 x 0.0484 HPV infected donor semen samples. This would result in 24241 (partner IUI cycles) and 1974 (donor IUI cycles) or a total of 26215 HPV compromised IUI cycles per year in Europe.

Only a systematic prospective study including an appropriate number of husband/partner or donor semen used in IUI should allow to establish a potential link between HPV infected semen and a low pregnancy rate in IUI. From cost/benefit point of view the cost of systematic HPV screening on IUI semen should be compared to the benefit of avoiding IUI treatments with low pregnancy rate prognosis.

## Summary

When reviewing the evidence it seems evident that determining the origin of the detected HPV DNA is the key to predict the impact of the HPV infection.

## Dividing cells

When the detected viral HPV DNA originates from a dividing cell, the detected HPV DNA is never infectious (dividing cells do not support virion production) and does not affect fertility, but the viral DNA can transform the dividing cell it resides in, which could in time lead to pre-cancer and cancer. High-risk HPV types have oncogenes (E6/E7) that produce oncogenic proteins that can transform dividing cells or even leads to blocking of the cell division of the early embryo. It always leads to a self-limiting HPV infection since no progeny virions can be made and the HPV life cycle ends.

## Non-dividing cells and spermatozoa

When the detected viral HPV DNA originates from non-dividing differentiated cells or from free virions, it is infectious and exerts its deleterious effects thru weakening or incapacitating the cells it resides in. For the already dying epithelial cells the new virions are released when the cells desquamate and allow to restart the HPV life cycle (HPV is not a lytic virus). In embryos the accumulation of newly formed virions in the syncytiotrophoblasts results in the weakening of the implantation bond with the endometrium and diminished energy uptake leads to miscarriage. For spermatozoa the binding or internalization of viral DNA also leads to a decrease in functionality of sperm (lower motility, DNA damage).

## Double trouble

Although in the majority of cases only one of the two HPV induced pathways is present, in almost one fifth of HPV infected women (18%) both the virion producing and HPV transforming pathway occurs at the same time. In most cases multiple HPV types are present (Depuydt et al., 2016a), but it is also possible that only one HPV type is detected (Fig. 3).

Independent of the number of HPV types detected, serial measurement of type specific viral load allows to identify the origin of the detected HPV DNA and makes it possible to assess the impact of the HPV infection for cancer screening or fertility.

Although HPV is not a lytic virus, harbouring its DNA will ultimately lead to cell death or immortality depending on the cell type.
